# Assessing the Impact of Land-Use Planning on the Atmospheric Environment through Predicting the Spatial Variability of Airborne Pollutants

**DOI:** 10.3390/ijerph16020172

**Published:** 2019-01-09

**Authors:** Longgao Chen, Long Li, Xiaoyan Yang, Yu Zhang, Longqian Chen, Xiaodong Ma

**Affiliations:** 1 School of Geography, Geomatics and Planning, Jiangsu Normal University, Xuzhou 221116, China; yangxy0705@163.com (X.Y.); yuzhang@jsnu.edu.cn (Y.Z.); xiaodgma@163.com (X.M.); 2 School of Environmental Science and Spatial Informatics, China University of Mining and Technology, Xuzhou 221116, China; chenlq@cumt.edu.cn; 3 Department of Geography, Earth System Science, Vrije Universiteit Brussel, Brussels 1050, Belgium

**Keywords:** land-use planning, environmental impact assessment, atmospheric quality, emission inventory, airborne pollution

## Abstract

As an important contributor to pollutant emissions to the atmosphere, land use can degrade environmental quality. In order to assess the impact of land-use planning on the atmosphere, we propose a methodology combining the land-use-based emission inventories of airborne pollutants and the long-term air pollution multi-source dispersion (LAPMD) model in this study. Through a case study of the eastern Chinese city of Lianyungang, we conclude that (1) land-use-based emission inventorying is a more economical way to assess the overall pollutant emissions compared with the industry-based method, and the LAPMD model can map the spatial variability of airborne pollutant concentrations that directly reflects how the implementation of the land-use planning (LUP) scheme impacts on the atmosphere; (2) the environmental friendliness of the LUP scheme can be assessed by an overlay analysis based on the pollution concentration maps and land-use planning maps; (3) decreases in the emissions of SO_2_ and PM_10_ within Lianyungang indicate the overall positive impact of land-use planning implementation, while increases in these emissions from certain land-use types (i.e., urban residential and transportation lands) suggest the aggravation of airborne pollutants from these land parcels; and (4) the city center, where most urban population resides, and areas around key plots would be affected by high pollution concentrations. Our methodology is applicable to study areas for which meteorological data are accessible, and is, therefore, useful for decision making if land-use planning schemes specify the objects of airborne pollutant concentration.

## 1. Introduction

Land resources are vital to support human survival and social development. In recent decades, however, they have been significantly influenced by human activities due largely to rapid demographic growth. Those activities directly related to land use include agriculture and urbanization, which have intensified changes in environmental quality on both regional and global scales [1]. Some land-use types are generally accompanied by pollutant emissions into water, soil, and the atmosphere, hence degrading the environmental quality [2,3] and even threatening human health [4,5,6]. Predicting the spatial variability of these airborne pollutants has, therefore, become an essential component of the assessment of the land-use impact on the environment. Land-use planning (LUP) (Table A1 in Appendix) describes the future land-use in terms of its pattern and distribution [7] in accordance with regional development strategies [8], especially at a city/county level. As such, environmental impact assessment of LUP (LUPEA) should evaluate the influence of future land-use on a regional environment [9]. 

In order to assess the land use and land-use planning’s impact on the atmospheric environment, a variety of assessment methodologies have been developed. Emission inventories prove useful for assessing atmospheric quality [10,11,12,13]. However, in general, emission inventories are mostly made for a specific industry or industrial category [14,15,16,17]. This means that the cost and time requirements for acquiring emission data are very high, particularly when emission inventories are used in strategic environmental assessment (SEA)—which has an even higher data demand. Land-use-based emission inventories, however, provide a feasible way to assess the overall pollutant emissions in a LUPEA applied to a moderate-scale geographical region (e.g., a typical Chinese city covering an area of thousands of square kilometers). 

Previous studies have used land-use regression models to characterize the variability of airborne pollutants [18,19,20,21,22], or dispersion models to predict and assess pollutant dispersion [23,24,25]. As the land-use regression models are statistically based, their results are less reliable for areas with a limited number of monitoring sites to measure the concentrations of airborne pollutants. This is, however, not the case for the airborne pollutant dispersion models—which are based on the investigation of pollutants, the impacts of regional meteorology, topography, and so on. 

In order to assess the impact of LUP implementation on the atmospheric environment, it is practical to use an airborne pollutant dispersion model if key factors contributing to pollution are determined. The long-term air pollution multi-source dispersion (LAPMD) model has, therefore, been developed, based on the prediction of pollutant dispersion in the reference year [26]. The assessment is expected to be improved if pollutant concentrations are calculated using pollutant inventories for the reference and individual target years.

In this study, we propose a new methodology combing land-use-based emission inventory and LAPMD model for assessing LUP impact on the atmospheric environment. The specific objectives of this study are (1) to establish a land-use-based emission inventory to support the assessment of the LUP impact on the atmosphere; (2) to develop a method for assessing the LUP impact on the atmospheric environment by combining a land-use-based emission inventory and the LAPMD model; and (3) to demonstrate the application of the methodology through a case study of Lianyungang, an eastern Chinese city in Jiangsu province.

## 2. Materials and Methods 

**Figure 1:**
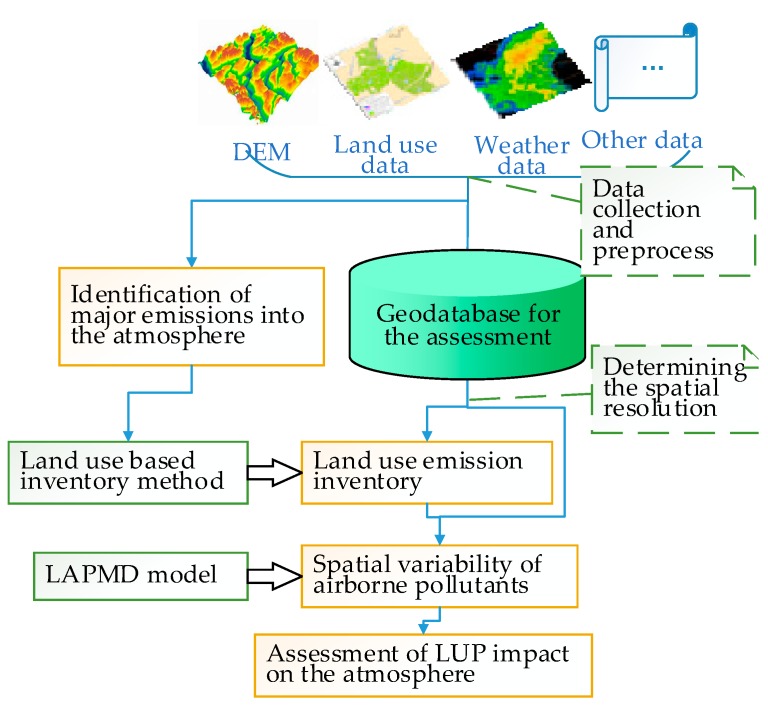
Flowchart for the land-use planning (LUP) atmospheric environment impact assessment.

An emission inventory of airborne pollutants can provide spatial source intensity for dispersion assessment. Emissions from individual land-use types in the reference and target years can be estimated using their respective inventories. By means of the LAPMD model, spatial variability of airborne pollutants in the reference and target years can be quantified, and the LUP impact on the atmospheric environment can be assessed. Figure 1 illustrates the procedure for our proposed assessment of LUP impact on the atmospheric environment. 

### 2.1. Compiling the Land-Use-Based Air Emissions Inventory

As emissions of airborne pollutants are closely associated with land use [27,28], we compiled an emission inventory based on land use. Since the assessment of regional LUP impact on the atmospheric environment serves the needs of making macro-level decisions, the precise locations of such emissions are considered unnecessary. It is acceptable to use coarse-resolution data (i.e., raster data with a large cell size), e.g., DEM or land-use data at hundreds of meters, for the environmental impact assessment of a large administrative region, e.g., a city or county in China which often covers a geographical area of thousands of square kilometers. 

Based on the land-use type, emission sources can be classified as three types, namely point, line, and area. For a city/county level LUP environmental assessment, point sources refer mainly to key plots for primary plants, line sources mostly include roads or rivers used for shipping, and area sources are residential land for both urban and rural populations. Because straw burning has been a widespread occurrence across China, particularly since the 2000s [29,30], agricultural land should be considered as a significantly important area source for assessing the land-use impact on the atmosphere.

Given the possibility and efficiency of data acquisition, we used the mass balance and emission factor-based methods [31,32,33,34] to obtain a land-use-based emission inventory. The mass balance method is based on the principle of mass conservation and thereby facilitates a ‘cradle-to-emissions’ approach [35]. The emission intensity of SO_2_ from land use can be obtained using Formula 1 according to the principle of the mass balance-based method:

(1) $$E=\frac{M_{SO_{2}}}{M_{s}}\times S\times \omega \times \varepsilon \times \left(1-\eta_{SO_{2}}\right)\times 10^{3}$$

where *E* is the emission intensity of SO_2_ (unit: kg), $M_{SO_{2}}$ the molecular weight of SO_2_ equaling 64, $M_{s}$ the molecular weight of sulfur equaling 32, *S* the rate of sulfur content in fuel, ω the fuel consumption (unit: ton), $\eta_{SO_{2}}$ the desulfurization rate, and ε the conversion rate of sulfur in fuel. 

The emission factor-based method calculates the emissions based on the average emission rate from a particular activity generating emissions [36]. For example, the emission intensity from transportation land is given by Formula 2:

(2) $$E_{i}=\sum P_{j}\times M_{j}\times EF_{i,j}\times 10^{-3}$$

where *E_i_* is the emission intensity of pollutant *i* (unit: kg), *P_j_* the holding quantity of vehicle *j*, *M_j_* the annual average running mileage of vehicle *j* (unit: km/per vehicle), *EF_i,j_* the emission factor of pollutant *i* from vehicle *j* (unit: g/km⋅per vehicle). 

After estimating the emission intensity, the spatial distribution of pollutant emissions for each year can be mapped in ArcGIS (Esri, Redlands, CA, USA), by assigning average emissions for individual land-use types (i.e., total emission intensity of pollutants for a land-use type divided by the number of raster cells for the land-use type) to their respective raster cells. 

### 2.2. Calculating the Annual Airborne Pollutant Concentration

The LAPMD model was used in this study to calculate the long-term land-use airborne pollutant multi-source concentration. This model can predict the annual dispersion of airborne pollutants based on a Gaussian dispersion model [26]. For the long-term dispersion of airborne pollutants, this model assumes that (1) the wind direction is divided into eight categories, each having a range of 45°, its frequency is equal for each fan-shaped area, and the pollutants are in the area and meet the regulation of Gaussian dispersion and (2) the source height value is the weighted average of different pollutant sources’ heights (the source height is the sum of the height of a pollutant source in relation to the ground surface and the height of the pollutant source’s bottom in relation to sea level, e.g., the source height for a 30-m-high chimney at 200 m above sea level is 230 m). Therefore, the LAPMD model can be described by the following equations:

(3) $$\int_{\alpha_{1}}^{\alpha_{2}}c\left(x,y\right)d_{y}=\frac{Q}{\sqrt{2\pi }\bar{u}\sigma_{y}\sigma_{z}}exp\left(-\frac{\Delta H+H}{2\sigma_{z}^{2}}\right)T$$

(4) $$c\left(x,y\right)=\left(\left(\frac{Q}{\sqrt{2\pi }\bar{u}\sigma_{y}\sigma_{z}}exp\left(-\frac{\Delta H+H}{2\sigma_{z}^{2}}\right)\right)/\frac{\pi x\left(\alpha_{2}-\alpha_{1}\right)}{180}\right)T$$

(5) $$\int_{0}^{\infty }\int_{\alpha_{1}}^{\alpha_{2}}c\left(x,y\right)d_{y}d_{x}=Q$$

where c(x,y) is the near ground average concentration of airborne pollutant in the grid with the position (x,y) in the fan-shaped area with the center of pollution source and angle from $\alpha_{2}$ to $\alpha_{1}$, $\sigma_{z}$ is the vertical dispersion parameter, $\sigma_{y}$ is the dispersion parameter of the grid with distance y from the source, Q is the source intensity of the pollutant in the grid, T is the attenuation parameter of pollutant, ΔH is the height of the pollutant source in relation to the ground surface (e.g., the height of a chimney), and H is the height of the pollutant source’s bottom in relation to sea level. A detailed description of the LAPMD model and the methods to extract the parameters can be found in our previous study [26]. In the methodology, the source intensity characterizes the land-use emission obtained in the inventory. All the spatial data are re-sampled to have an equal cell size for spatial analysis and calculation. After inventorying land-use emissions, parameters including the height of the pollutant source, elevation of the emission source, wind speed in each direction, dispersion parameters were put into the Matlab function of the LAPMD model. Then the annual land-use emission concentration maps at the same resolution will be obtained and imported into the geodatabase.

### 2.3. Assessing the LUP Impact on the Atmosphere

Long-term land-use airborne pollutant multi-source concentrations, which directly reflect the land-use impact on the atmospheric environment, were calculated using the LAPMD model for the reference and target years, respectively. The impact assessment can be performed by the spatial comparison of the airborne pollutant dispersion results between the reference and target years. In addition, the environmental friendliness of the land-use planning scheme can be evaluated through the overlay analysis of the land-use planning map with the concentrations map.

## 3. Case Study: Lianyungang, Eastern China

### 3.1. Study Area and Data

**Figure 2:**
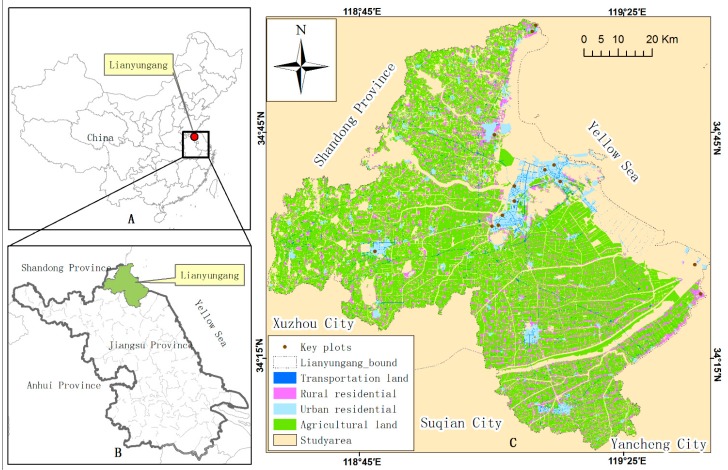
The location of Lianyungang (**A**,**B**) and its land-use map of 2010 (**C**). This map highlights the major land-use types and key plots emitting airborne pollutants in the study area.

To demonstrate the application of the proposed methodology, the land-use planning scheme of Lianyungang (2010–2020) was used to assess its LUP impact on the atmosphere for the reference year (2010) and target year (2020). As a coastal city in Jiangsu province in Eastern China (Figure 2), Lianyungang is characterized by a temperate monsoon climate with an annual precipitation of 920 mm and an average temperature of 26.5 °C in summer and of −0.4 °C in winter. Land-use types in this city include cropland, forest, water, transportation, commercial land, industrial, residential land, and typical coastal land-use classes such as tideland and saltern. A population of more than 4 million have led to drastic land use/cover changes in the region, especially since the 1980s. This has triggered considerable environmental challenges including atmospheric pollution. 

To predict the spatial variability of airborne pollutants and assess LUP impact on the atmospheric environment, the study area was limited to the entire geographical area of Lianyungang (i.e., both its urban and rural areas) and its adjacent areas (indicated in beige color in Figure 2). Spatial data used in the study include an improved 30-m ASTER GDEM (Advanced Spaceborne Thermal Emission and Reflection Radiometer Global Digital Elevation Model) dataset [37] as the ground height of the pollutant source, a land-use database of 2010, and the land-use planning scheme of Lianyungang for 2010–2020 obtained from local land management authorities. Daily meteorological data of 2010 (e.g., cloud cover, wind directions, and wind speeds) were freely available from the China Meteorological Data Sharing Service System. Data such as emissions of SO_2_ and PM_10_, attenuation parameter of pollutants, and pollutant source height (i.e., chimney height) were collected from local environmental authorities. Note that the pollutant source height is the sum of the ground height of the pollutant source and the chimney height.

### 3.2. Land-Use-Based Emission Inventory of Airborne Pollutants

#### 3.2.1. Classifying the Emission Sources 

Despite a wide range of emissions from land use into the atmosphere in Lianyungang, only primary pollutants that profoundly reduce environmental quality and human health are a primary concern to the public. As many industries in China continue to rely on the consumption of fossil fuels, particularly coal, due to the high cost of upgrading energy consumption, burning coal inevitably brings about emissions of particle matter (PM) and SO_2_, affecting the daily life of the regional residents. Given this fact, as well as consultancy from environmental experts, our assessment was focused on PM and SO_2_. 

PM_2.5_ emission has been widely considered as severe pollution in China, with the most serious effects in metropolises like Beijing [38] and Shanghai [39]. However, PM_2.5_ monitoring sites are sparse, and were even more so in the past [40]. In China, PM_2.5_ emission in all city-level administrative regions has been required to be monitored and published only since 2015 [41]—there was no PM_2.5_ data available in Lianyungang in 2010. Despite this, to some extent, the emissions of PM_10_ and SO_2_ which reflect that of PM_2.5_ [42], have been regularly presented in Lianyungang’s annual reports of environmental quality. Therefore, PM_10_ and SO_2_ were selected as significant airborne pollutants in the emission inventory for our assessment.

As mentioned in Section 2.1, key industrial plots with high emissions were classified as point sources, land used for vehicles as line sources, and residential and agricultural land as area sources, in our case study of Lianyungang. Locations of these land parcels were used to create features representing the pollutant sources in a GIS. All the features were imported into the assessment geodatabase and converted into raster data at 300-m resolution. Such a resolution was determined due to the trade-off between data accuracy and computing efficiency and also due to the nature of a strategic impact assessment. 

#### 3.2.2. Inventorying the Land-Use-Based Emissions 

The mass balance method was used to estimate the emission of SO_2_ from the point sources of key plots and the area sources of urban residential land (Formula 1) [43] except for the rural residential land where crop residues are largely burned for cooking. The conversion rate of sulfur in fuel was assigned as 80% for coal and as 100% for oil. The average desulfurization rate $\eta_{SO_{2}}$ of industrial plots were calculated using the coal consumption and SO_2_ emission data of some plants that we investigated. The $\eta_{SO_{2}}$ for area sources like urban residential land was assigned as 0 because no facilities were in place to reduce sulfur.

An emission inventory of PM_10_ from the point sources of key plots and the area sources of urban and rural residential land were built using the emission factor-based method. The emission factor value of point sources was assigned as the average rate derived based on the coal consumption and PM_10_ emission data of the investigated plants. The emission factor values of urban and rural residential land were derived from Kong et al. (2014) [31] (Table 1). Crop residues combustion in agricultural land produces various airborne pollutants including SO_2_ and PM_10_. The emission inventories of SO_2_ and PM_10_ from agricultural land were based on the agricultural crop residues combustion and emission factor values of SO_2_ and PM_10_ reported by Zhu et al. (2012) [32] (Table 1). 

Airborne pollutants from line sources were produced mainly by vehicles running on the road, either locally registered or coming from outside Lianyungang. Due to the absence of knowledge of the border-crossing vehicles in the city, we constrained the calculation of emissions to the locally registered vehicles. On the other hand, emissions from external vehicles may compensate those from local cars running out of the city to some extent. The annual average running mileage *M_j_* of a vehicle and the emission factor of the pollutant from a vehicle *EF_i,j_* were obtained from He et al., [33] and from Cai and Xie [34] respectively (Table 2). 

#### 3.2.3. Predicting the Emission Inventory Based on Land-Use Planning

Since a LUP scheme determines the future layout and arrangement of land parcels for a region [44], it helps to build emission inventories for years to come. In the LUP scheme of Lianyungang, a number of control and protection measures have been proposed to protect its environment. The factor values required in the inventory can be determined in light of the development of environmental protection technology and social economy, and the above-mentioned control and protection measures. To make the comparison easy to understand, the target year was assigned the same factor values as the reference year.

The emission in the target year was estimated in the same way as the reference year. We calculated the fuel consumption of key plots based on the estimated production-to-utilization ratio of fuel in the target year, the area of urban & rural residential land and the population in the corresponding areas, and the quantity of emission per capita in the reference year. We estimated the quantity of crop residue combustion based on the area of agricultural land, the quantity of emission per hm^2^ in the reference year, and the control of combustion in the target year, and estimated the fuel consumption of transportation land based on the estimation of holding vehicles and the pollutant emission standards of vehicles in the target year. Other factor values used in the target year considered in the inventory, such as the sulfur content rate of coal, the sulfur conversion rate of coal, and the annual average running mileage of individual vehicle types, were also the factor values in the reference year. Similarly, the emission inventory in the target year was obtained based on the layout of individual land-use types including transportation land, urban and rural residential, agricultural land, and key plots for primary plants.

### 3.3. Calculating the Annual Airborne Pollutant Concentration 

The annual spatial variability of airborne pollutants was mapped through the LAPMD model. The meteorology of the study area was investigated and used to calculate the parameters including $\sigma_{z}$, $\sigma_{y}$, and T. The wind was divided into eight directions, and the wind speed in each direction was calculated by averaging daily wind speeds in that direction in 2010. The average cloud amount and solar elevation angle in each wind direction were calculated based on the daily meteorological data and used to assess the dispersion parameters $\sigma_{z}$ and $\sigma_{y}$. Given the frequency of the wind direction, the source intensity was divided into eight parts. ΔH of urban and rural residential land was assigned as the average half height of the buildings respectively, ΔH of agricultural land as 0 m, and ΔH of key plots as the height of the chimney. H is the elevation of the pollutant source provided by ASTER GDEM data.

To execute the LAPMD model, the ASTER GDEM, the source intensity, and the height of the pollutant source were converted into a matrix and imported into Matlab 7.0 platform. A Matlab code representing the model was executed to obtain the matrix of airborne pollutant concentration of the study area which was later converted into raster data and imported into the geodatabase.

### 3.4. Assessing the LUP Impact on the Atmosphere

The long-term airborne pollutant concentration in the target year demonstrates the impact of LUP on the atmospheric environment. For better comparison, the concentration was classified into three levels according to the *Ambient Air Quality Standard in China (GB 3095-2012)* (AQSC) [45]. The environmental friendliness of the LUP scheme was assessed by an overlay analysis of the concentration maps and the LUP scheme. Then the area of individual land-use type scheme in the target year located at different concentration levels and the impact scopes of LUP on the atmospheric environment according to different levels were obtained. 

## 4. Results

The land-use-based emissions of Lianyungang in the reference year of 2010 and the target year of 2020 are presented in Table 1 and Table 2. Table 1 shows that significant changes in emissions from land-use point sources (key plots for primary plants) and area sources (urban and rural residential land and agricultural land) from 2010 to 2020. Despite increasing emissions of SO_2_ and PM_10_ from urban residential land, all the other point and area sources in Lianyungang would expect declining emissions. In total, both SO_2_ and PM_10_ emissions would decrease remarkably over the ten years. The SO_2_ emissions from transportation land (Table 2) are expected to decline as well. However, PM_10_ emissions show a slight rise from 2010 to 2020. In terms of individual vehicle types, only small cars and mini vehicles would produce increasing emissions of SO_2_ and PM_10_.

**Figure 3:**
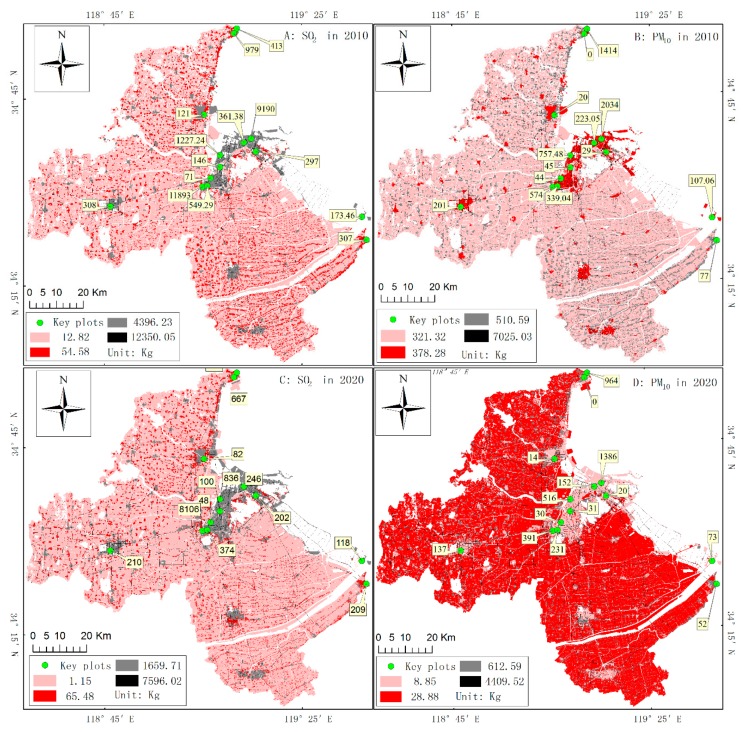
The source intensities of SO_2_ and PM_10_ in Lianyungang in 2010 and 2020: (**A**) and (**C**) are the concentrations of SO_2_ in 2010 and 2020, respectively; and (**B**) and (**D**) are the concentrations of PM_10_ in 2010 and 2020, respectively. The labels in the figures are the source intensity for key plots (unit: ton).

To build the geodatabase for estimating the concentrations of land-use-based emissions, the source intensities of SO_2_ and PM_10_ in 2010 and 2020 were mapped at 300 m resolution (Figure 3). Major emissions from key plots, located in the northern central part of Lianyungang, are labeled in Figure 3.

**Figure 4:**
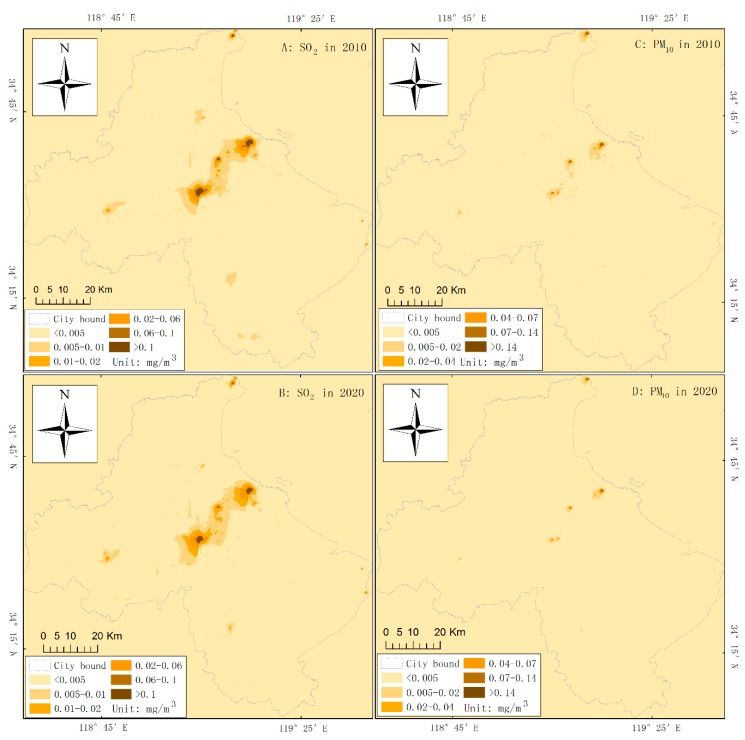
Classification maps of the SO_2_ and PM_10_ concentrations of Lianyungang in 2010 and 2020.

The spatial concentrations of SO_2_ and PM_10_ were mapped for 2010 and 2020 and classified into six levels of pollution (Figure 4). It is clear that the concentrations of SO_2_ and PM_10_ with high values (>0.005 mg/m^3^) are basically distributed in the city center and around key plots both in 2010 and 2020. There is a decrease in the average concentration of SO_2_ from 0.97 to 0.48 mg/m^3^ and of PM_10_ from 1.68 to 1.15 × 10^−3^ mg/m^3^ (Table 3). Notably, the areas of highest SO_2_ and PM_10_ concentration levels would decrease from 2010 to 2020.

## 5. Discussion

### 5.1. Land-Use-Based Atmospheric Emission Inventories

Studies on the land-use impact on the atmosphere reveal growing concerns about decreasing environmental quality which may threaten public health [47,48,49,50]. Among a variety of airborne pollutants [51], SO_2_, NOx, PM_10_, and PM_2.5_ were mostly investigated [11,52] since they are considered closely related to regional air quality [53]. In Asian countries, particularly in China and India, fine particles are a serious health threat. We understand that it would be important to consider PM_2.5_ for assessing the LUP impact on the atmosphere. However, we included PM_10_, rather than PM_2.5_ mainly due to the absence of the monitoring data of PM_2.5_ in 2010. In a LUPEA for a strategic purpose, the assessment should focus on the significant concerns with low cost and use indicators that are directly or easily available for a practicable purpose. In addition, previous studies have reported a high correlation between PM_10_ and PM_2.5_ emissions in China and beyond [54,55,56]. It is also reported that the contribution of PM_2.5_ to PM_10_ in the Central Black Sea in summer was 54% [57]. As such, to some extent, the estimation of PM_10_ reveals the concentration of PM_2.5_. These studies, on the other hand, provide an alternative method for estimating PM_2.5_ concentration based on PM_10_ data when PM_2.5_ is not regularly monitored.

In the emission inventories of Lianyungang in 2010 and 2020, data used in the assessment (e.g., land-use and land-use planning maps, annual socioeconomic statistical data, environmental quality reports) are usually quite accessible—this allows the methodology presented to be easily applied to other study areas, particularly those with restricted data acquisition. Equally importantly, a high spatial resolution is not required for an atmospheric assessment of a region covering thousands of square kilometers. 

According to Table 1 and Table 2, the decreases in SO_2_ and PM_10_ emissions from point sources from 2010 to 2020 illustrates the improvement of atmospheric quality based on the land-use planning for ten years. The significant decline in emissions from agricultural land might be attributed to the implementation of the no-straw-burning policy in China from 2010. The minimum utilization rate is estimated at 90% as the goal of Lianyungang in 2020 by the government of Lianyungang. The contrasting trends in SO_2_ and PM_10_ emissions from urban and rural residential lands over the 10 years (Table 1) can be explained by China’s rapid urbanization and economic development. A decrease in the rural population, as well as the area of rural residential land, predicted in the land-use planning scheme, would suggest lower SO_2_ and PM_10_ emissions from rural residential land in 2020 than in 2010. However, urban expansion would increase the emission of airborne pollutants including SO_2_ and PM_10_ during the 10 years—due to the daily activity of urban residents, such as heating and cooking.

The emission inventories for transportation land demonstrate how transportation land impacts the atmosphere (Table 2). In China, the quality of diesel and gasoline for vehicles has gradually been improved and car exhaust has therefore decreased. The emission factor in the target year (2020) was then estimated at ~60% of that in 2010 based on the regional upgrade regulation, the application of the corresponding laws and rules in China, and a Chinese national standard that limits exhaust gas [58]. Despite such improvements in the quality of diesel and gasoline, PM_10_ emissions are expected to rise slightly from 2010 to 2020—largely due to a growing demand for privately owned cars. In the LUP scheme of Lianyungang, the urban population would rise from 2.4 million to 2.86 million, whereas the rural population would decline from 2.6 million to 2.34 million. It is expected that population growth would bring about more vehicle use especially the small cars and mini vehicles since they are preferred in cities and towns. Despite fuel improvement, the rapid increase of the two vehicle types will lead to increasing SO_2_ and PM_10_ emissions to some extent (Table 2). Therefore, special attention should be given to optimizing the gasoline consumption of the two vehicle types and restricting their numbers. 

The SO_2_ and PM_10_ concentration maps in the reference and target years (Figure 3) highlight that the majority of key plots with very high emissions are mostly located in populated urban areas. Despite its low source intensity per cell (pixel), agricultural land makes a significant contribution to the emissions because it covers the largest portion of the study area. Except for point sources (key plots), transportation land—which forms a road network of the entire city—has the highest source intensities of the two pollutants in 2010 and 2020. This suggests that transportation land is one of the largest pollution sources in Lianyungang. 

### 5.2. Spatial Characteristics of Airborne Pollutant Concentration 

An industry-based environmental impact assessment considers the impact of every single project with detailed pollutant emissions [59,60,61]. Many of the models employed in environmental impact assessment simulating or predicting airborne pollutant require a variety of parameters and are run on an hourly or daily basis [62,63,64]. The high cost of data acquisition and calculation inevitably increase the difficulty of LUPEA due to the large geographic area, with numerous plants and a large variety of environmental indicators. As land-use planning is an annually based strategy to arrange the future land use, we consider it appropriate to employ the land-use-based emission inventory method and LAPMD model to assess the LUP impact of the atmospheric environment. In addition, this methodology also facilitates the calculation of the annual concentration of airborne pollutants at a low cost. It is an improvement in LUP impact assessment on the atmospheric environment, compared with our previous study in which the environmental friendliness of a Chinese county’s LUP was assessed only using the pollutant concentration in the reference year [26]. Moreover, the environmental friendliness of urban and rural residential land planning was obtained through an overlay analysis of the emission concentration maps and the urban and rural land planning map (Table 4). 

The decreases in SO_2_ concentration from 0.97 to 0.48 and in PM_10_ concentration from 1.68 to 1.15 × 10^−3^ mg/m^3^ (Table 3) in 2010 and 2020 demonstrate that the impact of LUP on the atmosphere is expected to lessen, which is also proven by the shrinking areas of the highest concentration levels for both SO_2_ and PM_10_ from 2010 to 2020. We consider that such a weakening impact is a result of the implementation of the land-use planning in Lianyungang. Considering the maximum concentration threshold for the second level in the AQSC, the area with PM_10_ concentrations over 0.07 mg/m^3^ would decrease more than the area with SO_2_ concentrations over 0.06 mg/m^3^ (Figure 4, Table 3). In fact, the rapid urbanization in Lianyungang will lead to population aggregation in the city center, contributing to higher airborne emissions by means of heating, cooking, and running vehicles (especially small cars and mini vehicles). Otherwise, the air pollution will be expected to decrease more than predicted, owing to the environmental measures in land-use planning.

### 5.3. Evaluating the Environmental Friendliness of LUP

The concentration maps are also useful in evaluating the environmental friendliness of LUP. By overlaying the concentration maps with the vector data representing the newly planned urban and rural land in GIS, we obtained the affected area at different levels (Table 4). As most of the newly planned rural land (>95%) has very low SO_2_ and PM_10_ concentrations (<0.005 mg/m^3^). it is understood that that the overall impact of the layout of new rural residential land is environmentally friendly. A small part of that newly planned urban land with SO_2_ concentrations <0.005 mg/m^3^ in 2020 (69.43%) reveals noticeable differences in emissions from urban land and rural land, with the former releasing more SO_2_. The overlay analysis also reveals that all the area has PM_10_ concentrations <0.04 mg/m^3^ both in 2010 and 2020 and that the PM_10_ pollution would have little negative influence on the newly planned residential land. In examining the newly planned urban and rural lands, we note that the new urban residential land will be more affected by air pollution than new rural residential land, and that specific efforts are therefore needed to control emissions for these two types of newly planned land. 

## 6. Conclusions

Land use can increase the emissions of airborne pollutants into the atmosphere and degrade environmental quality; it is, therefore, essential to consider the impact on the atmospheric environment during land-use planning. In this study, we proposed a methodology combining the land-use-based emission inventories of airborne pollutants and the LAPMD model to assess the land-use planning atmospheric impact. Land-use-based emission inventories prove effective and feasible in collecting the overall pollutant emissions at a low cost when compared with the industrially based method, and a moderate spatial resolution is appropriate to a city-scale LUPEA. Through the land-use-based emission inventories, the LAPMD model allows a characterization of the spatial variability directly reflecting the impact of the LUP on the atmosphere. The environmental friendliness of LUP can also be assessed by overlaying the concentration maps with the newly planned urban and rural land map.

In the case study of Lianyungang, some land-use types such as urban residential and transportation land are related to higher emissions. The overall decreases in average SO_2_ and PM_10_ concentrations from the reference year of 2010 to the target year of 2020 suggest that improved atmospheric quality would be expected thanks to the implementation of land-use planning. High concentrations are mostly distributed in the city center and around key plots. There is, therefore, a concern that urban population in the affected areas will be exposed to high pollution concentrations. Although the overlay analysis reveals that the layout of newly planned urban and rural lands is environmentally friendly, emission reduction measures should be in place for the newly planned urban lands located in the areas of high SO_2_ and PM_10_ concentrations (>0.005 mg/m^3^). 

The methodology proposed in this study highlights the assessment of the land-use planning impact on the atmosphere from a land-use point of view. It promises a low cost of data acquisition and investigation and is applicable to other areas for which data availability is restricted and, in the case of similar dispersion, to the studies which focus on airborne pollutants such as NOx and PM_2.5_. Moreover, by means of the correlation of the exploration of PM_10_ and PM_2.5_ found in other studies, it is possible to assess PM_2.5_ based on the PM_10_ data. Findings from this study are helpful for decision making if land-use planning schemes specify the objectives of airborne pollutant concentration explicitly.

## Figures and Tables

**Table 1:** Emission inventories of SO_2_ and PM_10_ from land-use point and area sources in Lianyungang.

Year	Emission Source Types	Emission Factor of SO_2_ (kg/ton)	Emission of SO_2_		DesulfuriZation Rate	Emission Factor of PM_10_ (kg/ton)	Emission of PM_10_	
			(ton)	(%)			(ton)	(%)
2010	Key plot	-	26,036.38	74.1	0.6	2.97	5864.63	23.21
	Urban residential	-	8026.77	22.85	0	0.15	42.81	0.17
	Rural residential	0.4	478.5	1.36	-	3.74	4476.55	17.72
	Agricultural land	0.4	593.8	1.69	-	10	14,884	58.9
	Total		35,135.45	100			25,267.99	100
2020	Key plot	-	17,746.32	63.82	0.6	2.97	3997.31	41.42
	Urban residential	-	9565.23	34.4	0	0.15	51.02	0.53
	Rural residential	0.4	430.65	1.55	-	3.74	4028.9	41.74
	Agricultural land	0.4	62.8	0.23	-	10	1574.25	16.31
	Total		27,805	100			9651.48	100

**Table 2:** Atmospheric emission inventory of vehicle-based transportation land sources in Lianyungang.

Year	Vehicle Types	Emission Factor of SO_2_ (kg/ton)	Emission of SO_2_		Emission Factor of PM_10_ (kg/ton)	Emission of PM_10_	
			(ton)	(%)		(ton)	(%)
2010	1. passenger vehicle						
	Large vehicle	0.05	786.52	5.08	0.02	314.61	3.57
	Medium vehicle	0.01	153.69	0.99	0.02	307.37	3.49
	Small car	0.01	2118.24	13.68	0.02	4236.48	48.11
	Mini vehicle	0.01	98.59	0.64	0.02	197.19	2.24
	2. truck						
	Heavy truck	0.10	8918.00	57.61	0.02	1783.60	20.26
	Medium truck	0.05	1305.68	8.43	0.02	522.27	5.93
	Light truck	0.01	375.40	2.43	0.02	750.80	8.53
	Mini truck	0.01	2.18	0.01	0.02	4.36	0.05
	3. tricar	0.05	1721.37	11.12	0.02	688.55	7.82
	Total		15,479.67	100	0.02	8805.24	100
2020	1. passenger vehicle						
	Large vehicle	0.030	490.79	4.29	0.012	196.32	2.16
	Medium vehicle	0.006	95.90	0.84	0.012	191.80	2.12
	Small car	0.006	3029.09	26.46	0.012	6058.17	66.81
	Mini vehicle	0.006	140.99	1.23	0.012	281.98	3.11
	2. truck						
	Heavy truck	0.060	5564.83	48.62	0.012	1112.97	12.27
	Medium truck	0.030	814.74	7.12	0.012	325.90	3.59
	Light truck	0.006	234.25	2.05	0.012	468.50	5.17
	Mini truck	0.006	1.36	0.01	0.012	2.72	0.03
	3. tricar	0.030	1074.13	9.38	0.012	429.65	4.74
	Total		11,446.08	100	0.012	9068.01	100

Note: Vehicles are classified in light of the *2011 statistical yearbook of Lianyungang* [46].

**Table 3:** Areas of SO_2_ and PM_10_ concentrations at different levels in 2010 and 2020.

Value (Unit: mg/m^3^)	Area				Value (Unit: mg/m^3^)	Area			
	PM_10_ in 2010		PM_10_ in 2020			SO_2_ in 2010		SO_2_ in 2020	
<0.005	16,630.5	99.48%	16,678.51	99.77%	<0.005	16,341.66	97.75%	16,301.61	97.51%
0.005–0.02	73.52	0.44%	30.89	0.18%	0.005–0.01	270.61	1.62%	282.57	1.69%
0.02–0.04	7.12	0.04%	4.05	0.02%	0.01–0.02	51.78	0.31%	84.54	0.51%
0.04–0.07	2.82	0.02%	2.16	0.01%	0.02–0.06	34.79	0.21%	35.76	0.21%
0.07–0.14	1.92	0.01%	0.9	0.01%	0.06–0.1	6.74	0.04%	5.51	0.03%
>0.14	1.17	0.01%	0.54	0.00%	>0.1	11.48	0.07%	7.05	0.04%
Average value (Unit: 10^−3^ mg/m^3^)	0.97		0.48			1.68		1.15	

**Table 4:** Distribution of the SO2 and PM10 concentrations in the newly planned urban and rural land by 2020 in the LUP scheme.

Land Scheme	Value	Area (hm^2^)				Value	Area (hm^2^)			
	(mg/m^3^)	SO_2_ in 2010		SO_2_ in 2020		(mg/m^3^)	PM_10_ in 2010		PM_10_ in 2020	
Newly planned rural land	<0.005	1103.24	96.63%	1098.55	96.22%	<0.005	1132.93	99.23%	1139.31	99.79%
	0.005–0.01	32.3	2.83%	32.79	2.87%	0.005–0.02	8.82	0.77%	2.43	0.21%
	0.01–0.02	3.78	0.33%	4.19	0.37	0.02–0.04	0.00	0.00%	0.00	0.00%
	0.02–0.06	0	0.00%	3.78	0.33	0.04–0.07	0.00	0.00%	0.00	0.00%
	0.06–0.1	0	0.00%	2.33	0.20	0.07–0.14	0.00	0.00%	0.00	0.00%
	>0.1	2.43	0.21%	0.11	0.01	>0.14	0.00	0.00%	0.00	0.00%
Newly planned urban land	<0.005	10,599.37	80.67%	9123.03	69.43%	<0.005	12,876.68	98.00%	13,083.84	99.57%
	0.005–0.01	2255.27	17.16%	3223.11	24.53%	0.005–0.02	247.46	1.88%	56.12	0.43%
	0.01–0.02	199	1.51%	688.26	5.24%	0.02–0.04	15.81	0.12%	0.00	0.00%
	0.02–0.06	82.04	0.62%	100.6	0.77%	0.04–0.07	0.00	0.00%	0.00	0.00%
	0.06–0.1	2.18	0.02%	1.83	0.01%	0.07–0.14	0.00	0.00%	0.00	0.00%
	>0.1	2.11	0.02%	3.12	0.02%	>0.14	0.00	0.00%	0.00	0.00%

## Appendix

**Table A1:** Full forms and description of the acronyms.

Acronym	Full Form	Description	Reference(s)
LUP	Land-use planning	The process for the spatio-temporal arrangement and allocation of land recourses in accordance with the principles of sustainable land-use.	T. Tang, Zhu, & Xu, (2007) [7]; Z. Tang, Bright, & Brody, (2009) [8]
SEA	Strategic Environmental Assessment	The process for evaluating the environmental consequences of proposed policy, plan or programme (PPP) and its alternatives in order to ensure they are fully considered and appropriately addressed at the earliest suitable stage of the decision-making process.	Chen et al., (2014) [44]
LUPEA	Land-use planning environmental impact assessment	The application of SEA in LUP is known as LUPEA. It is a process for assessing the environmental impact of LUP including before, during and after the implementation.	Chen, Yang, Chen, & Li, (2015) [9]; Chen et al., (2014) [44]
LAPMD	Long-term air pollution multi-source dispersion model	A model to spatially estimate the annual concentration of atmospheric pollutants, it was established based on the Gaussian diffusion model.	Chen, Yang, & Kang, (2012) [26]
